# Big Multiple Sclerosis Data network: an international registry research network

**DOI:** 10.1007/s00415-024-12303-6

**Published:** 2024-04-01

**Authors:** Anna Glaser, Helmut Butzkueven, Anneke van der Walt, Orla Gray, Tim Spelman, Chao Zhu, Maria Trojano, Pietro Iaffaldano, Mario A. Battaglia, Giuseppe Lucisano, Sandra Vukusic, Irena Vukusic, Romain Casey, Dana Horakova, Jiri Drahota, Melinda Magyari, Hanna Joensen, Luigi Pontieri, Frederik Elberling, Pernilla Klyve, Elena Flavia Mouresan, Lars Forsberg, Jan Hillert

**Affiliations:** 1 Department of Clinical Neuroscience, Karolinska Institute, Stockholm, Sweden; 2 Department of Neuroscience, Central Clinical School, Monash University, Melbourne, VIC Australia; 3 Department of Neurology, The Alfred Hospital, Melbourne, VIC Australia; 4 South Eastern Health and Social Care Trust, Belfast, UK; 5 School of Medicine, University “Aldo Moro”, Bari, Italy; 6 Department of Translational Biomedicine and Neurosciences, DiBraiN University of Bari Aldo Moro, Bari, Italy; 7 Research Department, Italian Multiple Sclerosis Foundation, Genoa, Italy; 8 Department of Life Sciences, University of Siena, Siena, Italy; 9 Center for Outcomes Research and Clinical Epidemiology-CORESEARCH, Pescara, Italy; 10 Service de Neurologie, sclérose en plaques, pathologies de la myéline et neuro-inflammation, Hospices Civils de Lyon, Hôpital Neurologique Pierre Wertheimer, 69677 Bron, France; 11 INSERM 1028 et CNRS UMR 5292, Observatoire Français de la Sclérose en Plaques, Centre de Recherche en Neurosciences de Lyon, 69003 Lyon, France; 12 Université de Lyon, Université Claude Bernard Lyon 1, 69000 Lyon, France; 13 Eugène Devic EDMUS Foundation Against Multiple Sclerosis, State-Approved Foundation, 69677 Bron, France; 14 Department of Neurology and Center of Clinical Neuroscience, First Faculty of Medicine Charles University and General University Hospital in Prague, Prague, Czech Republic; 15 IMPULS Endowment Fund, Prague, Czech Republic; 16 Department of Neurology, Danish Multiple Sclerosis Center, Copenhagen University Hospital Rigshospitalet, 2100 Copenhagen, Denmark; 17 The Danish Multiple Sclerosis Registry, Copenhagen University Hospital, Rigshospitalet, Copenhagen, Denmark

**Keywords:** Multiple sclerosis, Patient registries, Patient data network, Real-world evidence

## Abstract

**Background:**

The Big Multiple Sclerosis Data (BMSD) network (https://bigmsdata.org) was initiated in 2014 and includes the national multiple sclerosis (MS) registries of the Czech Republic, Denmark, France, Italy, and Sweden as well as the international MSBase registry. BMSD has addressed the ethical, legal, technical, and governance-related challenges for data sharing and so far, published three scientific papers on pooled datasets as proof of concept for its collaborative design.

**Data collection:**

Although BMSD registries operate independently on different platforms, similarities in variables, definitions and data structure allow joint analysis of data. Certain coordinated modifications in how the registries collect adverse event data have been implemented after BMSD consensus decisions, showing the ability to develop together.

**Data management:**

Scientific projects can be proposed by external sponsors via the coordinating centre and each registry decides independently on participation, respecting its governance structure. Research datasets are established in a project-to-project fashion and a project-specific data model is developed, based on a unifying core data model. To overcome challenges in data sharing, BMSD has developed procedures for federated data analysis.

**Future perspectives:**

Presently, BMSD is seeking a qualification opinion from the European Medicines Agency (EMA) to conduct post-authorization safety studies (PASS) and aims to pursue a qualification opinion also for post-authorization effectiveness studies (PAES). BMSD aspires to promote the advancement of real-world evidence research in the MS field.

## Background

**Fig. 1:**
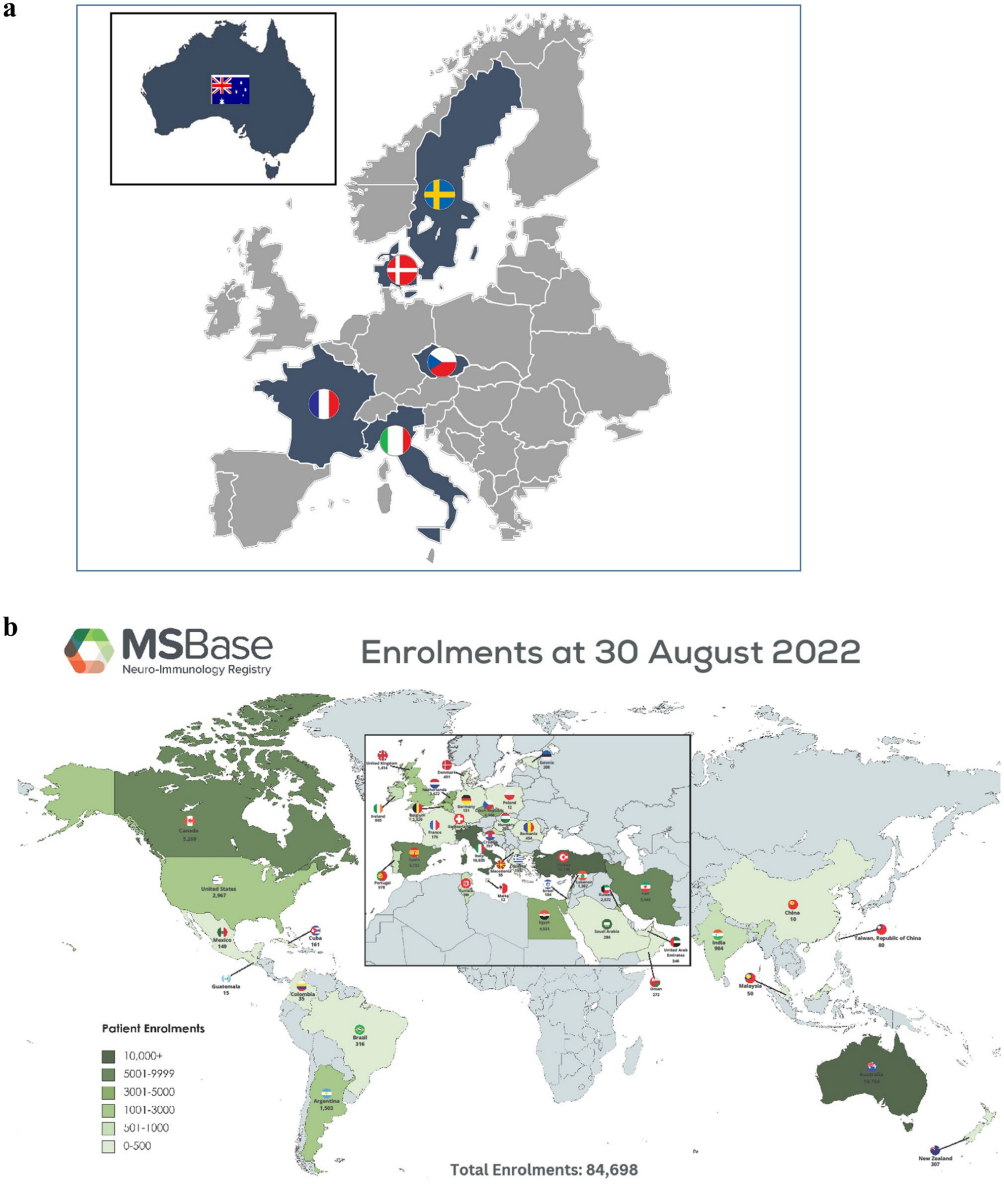
**a** Map of MS registries. The national MS registries of Czech Republic, Denmark, France, Italy, Sweden and the international MSBase with home in Australia. **b** Map of MSBase registries

The emergence of disease-modifying treatments (DMTs) for multiple sclerosis (MS) in the 1990s made it clear that longitudinal and structured collection of clinical data from MS care, including treatments and outcomes, would be required to assess long-term effectiveness and safety. Consequently, some pre-existing MS registries and databases initiated the collection of treatment information, such as in Denmark [1] and France [2], whereas new national MS registries were started in other countries, including Italy [3] and Sweden [4]. Moreover, the establishment of MSBase, an international database collaboration, was initiated with the purpose of creating a global data collection platform regardless of nationality [5]. With time, it became clear that these MS registries successfully managed to collect high-quality longitudinal clinical information on large patient cohorts, contributing to a growing body of scientific literature of real-world evidence (RWE) in MS [6]. These studies focus on the epidemiology of MS including incidence, prevalence, mortality, natural disease course and time trends. Importantly, registry data contributes to pharmacoepidemiology which includes comparative effectiveness and socioeconomic studies. Following that, it became evident that RWE in MS could have potential benefits for marketing authorization holders (MAHs) and regulators. Simultaneously, the European Medicines Agency (EMA) and the U.S. Food and Drug Administration (FDA) have released guidelines outlining how disease registries can serve as a foundation for regulatory determinations. Notably, EMA has identified MS as a disease in which this approach could be pioneered. The establishment of the Big Multiple Sclerosis Data (BMSD) network (https://bigmsdata.org) in 2014 was made possible by an initial grant from Biogen. Since 2019, pharma companies in the MS field have been approached and asked for their willingness to support the current activities and development of BMSD. Six pharma have supported BMSD for 3 years or more: Biogen, Bristol-Myers-Squibb, Merck, Novartis, Roche, and Sanofi. For 2023, five pharma supported BMSD. At the start, the network included the national MS registries of Denmark, France, Italy, and Sweden as well as the international MSBase registry. The national MS registry of the Czech Republic [7], previously represented by MSBase, now participates as an individual registry within the network, bringing the participating registry number to six (Fig. 1a, b). The BMSD network is made up of well-developed registries, with reasonable coverage of local MS patients, providing a reliable framework for the network and containing data from a large number of people with MS (Table 1). Each of these registries is well established as data sources for multiple scientific publications over the years (see Table 2). The plan for the future is to include more registries in the network given that they meet the expected criteria for BMSD.

**Table 1:** BMSD MS registries

Country	MS registry	Home page	Number of people with MS included	Estimated coverage of prevalent MS population
Czech Republic	ReMuS	http://www.multiplesclerosis.cz	21,500 January 2024	80%
Denmark	DMSR	http://www.dmsr.dk	33,142 February 2024	95%
France	OFSEP	http://www.ofsep.org	81,325 December 2023	50%
Italy	IMSREg	https://registroitalianosm.it/en/	87,045 February 2024	60%
Sweden	SMSreg	https://www.neuroreg.se/multipel-skleros/	23,567 February 2024	85%
International	MSBase	https://www.msbase.org/	97,263^a^ February 2024	Variable depending on the contributing centres, e.g., around 40% in Australia, 20% in Turkey and 95% in Kuwait

^a^The Italian and the Czech Republic’s MS registries operate as independent entities but some of their centres also contribute data to MSBase. Any overlap is removed prior to the initiation of a study so that patients only contribute once

**Table 2:** Three selected recent publications from each of the BMSD registries with links to complete publication lists

Registry/country	Full publication lists available at	Selected recent publications
ReMuS Czech Republic	http://www.multiplesclerosis.cz/en/publikace-z-dat-registru/	Dominika Stastna, Jiri Drahota, Michal Lauer et al. The Czech National MS Registry (ReMuS): data trends in multiple sclerosis patients whose first disease-modifying therapies were initiated from 2013 to 2021. Biomed Pap Med Fac Univ Palacky Olomouc Czech Repub 2023; 167:XX Hrnciarova T, Drahota J, Spelman T, et al. Does initial high efficacy therapy in multiple sclerosis surpass escalation treatment strategy? A comparison of patients with relapsing–remitting multiple sclerosis in the Czech and Swedish national multiple sclerosis registries. Mult Scler Relat Disord. 2023 Aug;76:104803. 10.1016/j.msard.2023.104803 Hradilek P, Zapletalova O, Hanulikova P et al. Is breastfeeding in MS harmful or not? An answer from real-world Czech data. Mult Scler Relat Disord
DMRS Denmark	http://www.dmsr.dk/publications.html	Wandall-Holm MF, Holm RP, Heick A, Langkilde AR, Magyari M. Risk of T2 lesions when discontinuing fingolimod: a nationwide predictive and comparative study. Brain Commun. 2024 Jan 2;6(1):fcad358 Magyari M, Joensen H, Kopp TI, Pontieri L, Koch-Henriksen N. Changes in prognosis of the Danish multiple sclerosis population over time. Mult Scler. 2022 Jul 13 Magyari M, Koch-Henriksen N Quantitative effect of sex on disease activity and disability accumulation in multiple sclerosis. Journal of Neurology, Neurosurgery, and Psychiatry. 2022; 93:716–722
OFSEP France	http://www.ofsep.org/en/publications-en	Rollot F, Uhry Z, Dantony E, et al. Comparison of 2 methods for estimating multiple sclerosis-related mortality. Neurology. 2023 Dec 12;101(24):e2483–e2496 Gavoille A, Rollot F, Casey R, et al. Investigating the long-term effect of pregnancy on the course of multiple sclerosis using causal inference. Neurology. 2023 Mar 21;100(12):e1296–e1308 Lebrun-Frénay C, Rollot F, Mondot L, et al. Risk factors and time to clinical symptoms of multiple sclerosis among patients with radiologically isolated syndrome. JAMA Netw Open. 2021 Oct 1;4(10):e2128271. 10.1001/jamanetworkopen.2021.28271. PMID: 34633424
IMSREg Italy	http://www.registroitalianosm.it/en/index.php?page=publications	Iaffaldano P, Portaccio E, Lucisano G, et al. Multiple sclerosis progression and relapse activity in children. JAMA Neurol. 2024 Jan 1;81(1):50–58. 10.1001/jamaneurol.2023.4455 Portaccio E, Fonderico M, Iaffaldano P, et al. Disease-modifying treatments and time to loss of ambulatory function in patients with primary progressive multiple sclerosis. JAMA Neurol. 2022 Sep 1;79(9):869–878. 10.1001/jamaneurol.2022.1929 Portaccio E, Bellinvia A, Fonderico M, et al. Progression is independent of relapse activity in early multiple sclerosis: a real-life cohort study. Brain. 2022 Aug 27;145(8):2796–2805. 10.1093/brain/awac111
SMSreg Sweden	http://www.neuroreg.se/media/yn1j540i/ms-registret-publikationslista-2003-2022.pdf	Spelman T, Magyari M, Butzkueven H, et al. Predictors of treatment switching in the Big Multiple Sclerosis Data Network. Front Neurol. 2023 Dec 22;14:1274194. 10.3389/fneur.2023.1274194. 2023. PMID: 38187157 He A, Manouchehrinia A, Glaser A, et al. Premorbid sociodemographic status and multiple sclerosis outcomes in a universal health care context. JAMA Netw Open. 2023 Sep 5;6(9):e2334675. 10.1001/jamanetworkopen.2023.34675 Longinetti E, Englund S, Burman J et al. Trajectories of cognitive processing speed and physical disability over 11 years following initiation of a first multiple sclerosis disease-modulating therapy. J Neurol Neurosurg Psychiatry. 2024 Jan 11;95(2):134–141. 10.1136/jnnp-2023-331784. PMID: 37558400
MSBase International	http://www.msbase.org/data-and-findings/peer-reviewed-journal-articles/	Kalincik T, Sharmin S, Roos I, et al. Comparative effectiveness of autologous hematopoietic stem cell transplant vs fingolimod, natalizumab, and ocrelizumab in highly active relapsing–remitting multiple sclerosis. JAMA Neurol. 2023 Jul 1;80(7):702–713. 10.1001/jamaneurol.2023.1184. PMID: 37437240; PMCID: PMC10186210 Diouf I, Malpas CB, Sharmin S, et al. Effectiveness of multiple disease-modifying therapies in relapsing–remitting multiple sclerosis: causal inference to emulate a multiarm randomised trial. J Neurol Neurosurg Psychiatry. 2023 Jul 6:jnnp-2023–331499. 10.1136/jnnp-2023-331499. Epub ahead of print. PMID: 37414534 Sharmin S, Roos I, Simpson-Yap S, et al. The risk of secondary progressive multiple sclerosis is geographically determined but modifiable. Brain. 2023 Jun 27:awad218. 10.1093/brain/awad218. Epub ahead of print. PMID: 37369086

In its early phase, BMSD mapped the member registry datasets to a minimum data set and common data model (CDM) of variables, definitions and data structure and addressed the many formal challenges of data sharing that include ethical, legal and governance aspects. As a result, data sharing and pooling were demonstrated to be feasible, leading to the execution of a series of demonstrator projects utilizing pooled data. This resulted in the publication of a number of papers thus far, with a specific focus on the long-term effectiveness of DMTs, progressive MS, and the analysis of discontinuation patterns over time [8–11].

The network’s aspiration is to harness the data from over 250,000 MS patients provided by the participating registries, thereby creating an unparalleled sample size for collaborative analysis. This vast amount of data holds the potential to yield valuable insights and findings that would otherwise be unattainable. This may be especially valuable in the context of uncommon events such as rare serious adverse events (SAEs) but also for the analyses of the study of subgroups of patients under-represented in clinical trials (e.g. children and the elderly, or patients with specific comorbidities such as cancer). Over the past decades, MAHs and regulatory organisations, such as EMA, have begun to recognise registries as potentially useful data source, especially in the context of post-authorisation safety (PASS) and effectiveness (PAES) studies. BMSD is in the process of seeking an EMA qualification opinion for PASS and has received Scientific Advice and a letter of support from EMA (https://www.ema.europa.eu/en/documents/other/letter-support-performing-registry-based-post-authorisation-safety-studies-pass-multiple-sclerosis-ms-using-data-big-ms-data-network-bmsd_en.pdf). All BMSD partners are currently contributing to PASS projects, and a qualification opinion would empower BMSD to take further responsibility for such regulator demanded studies.

## Data collection

The data collected by the respective registries and their governance frameworks are a result of many years of development and has evolved by consensus within each registry organization. While the registries operate separately, they have all developed models of long-term success. Despite the independent nature of data collection, the similarities between data collected within the core dataset are striking. These similarities reflect a common aim to include variables that hold clinical significance. Additionally, all registries have developed high-quality data visualisation tools to support their data entry modules, which support neurologists in daily care and aid decisions related to individual patients, as well as providing data for research and other types of studies. Table 3 shows a core set of variables available from all registries. Although the data collection within each registry is subject to its respective governance bodies, common needs within certain collaborations have occasionally prompted agreement to include additional data in the collection, for instance during the COVID-19 pandemic.

**Table 3:** Description of core variables available from BMSD registries

	Variable	Czech Republic	Denmark	France	Italy	Sweden	MSBase
Demographics	Date of birth^a^	y	y	Month and year of birth	y	y	Month and year of birth
	Country of birth	y	Linkage	y	y	Linkage	y
	Date of death (if applicable)	y	Updated once a year	y	y	y	y
	Sex	y	y	y	y	y	y
Diagnosis	Date of MS onset	y	y	y	y	y	y
	Date of MS diagnosis	y	y	y	y	y	y
	MS criteria	y	y	y	y	y	y
	MS type (CIS, RR, SP, PP)	y	y	y	y	y	y
Disease specific information	Family history of MS	y	Linkage	y	y	Linkage	y
	MS attacks including dates of onset	y	y	y	y	y	y
	MS attack type	y	n	y	y	y	y
	CSF OCB	y	y	y	y	y	y
	CSF IgG index	y	y	y	y	y	y
	MRI supporting MS	y	y	y	y	y	y
	EDSS all	y	y	y	y	y	y
Co-morbidities		y	Linkage	y^a^	y	Linkage	y
DMT treatment	DMT start date	y	y	y	y	y	y
	DMT stop date and reason for stop	y	y	y	y	y	y

Linkage to public registries can be performed in Denmark and Sweden

^a^Retrospectively: cancer and PML; prospectively: SAE (since 2017)

The support of EMA and FDA to use patient registries as a basis for post-approval studies, mainly for PASS, has prompted safety to become a strong focus of BMSD in recent years. Accordingly, together with a group of pharma representatives from Biogen, Bristol Myers Squibb, Merck, Novartis, Roche, and Sanofi, BMSD has developed a *core protocol for PASS*. This includes a core dataset that all MS registries taking part in BMSD will be expected to follow, importantly including reported SAEs. SAEs are routinely collected in all the contributing MS registries in connection with specific treatments and can potentially be compared to unexposed groups. It is important to highlight that the SAE information collected by the individual registries has already been directly reported to the corresponding medicine product agencies (MPA) through parallel mechanisms. As a result, this data is classified as secondary and is not subject to pharmacovigilance reporting requirements. That responsibility remains with the treating physicians as legally specified, but the registry IT platforms may indeed help identify SAEs and alert physicians to report in a routine manner. Information on the SAEs aims to be classified using MedDRA terms when possible by the registries and efforts to put this in place are ongoing. Although all the registries collect some pregnancy outcome variables, some, like the Scandinavian countries, receive this information by linkage to public registries.

All BMSD registries are designed to collect SAEs. As an example, progressive multifocal leukoencephalopathy (PML), which is associated with some MS treatments, is expected to be reported. Important data items that could either improve the data collection or be of importance for risk stratification, such as lymphocyte counts (which are associated with the risk of PML during dimethyl fumarate exposure) are reported by some registries and could be relevant to propose as new data items for the other registries. Relevant new data items will be adopted over time. In fact, all BMSD registries have recently improved their collection of SAEs by adding specific questions answered at each visit/contact regarding malignancies, non-melanoma skin cancers and severe or immunosuppression-related infections (exemplified by herpes zoster). This shows that BMSD can, and in a coordinated fashion, adopt a relevant change in data collection in response to the needs of safety studies.

Typically, BMSD registries are not expected to collect non-serious adverse events, such as gastrointestinal symptoms associated with some DMTs. It is uncertain to what extent non-serious but clearly treatment-related events can be efficiently collected even if considered relevant, as the burden of data collection within contributing centres is considerable.

## Data management and analysis

BMSD is a collaboration of independent MS registries and clinical outcomes databases designed to address a wide range of clinical, pharmaceutical and epidemiological research questions using a flexible CDM). Datasets are set up in a project-to-project fashion for which a project-specific statistical analysis plan is developed, based on a core CDM. Furthermore, BMSD has the facilities to securely manage and store patient-level data and also intends to set up a repository of data counts on key variables which will be updated periodically.

The large collection of data from all the BMSD registries creates a very rich combined dataset of over 250,000 patient records. In the initial studies, datasets from the respective registries were merged into a common database before analysis. Such pooling, when possible, greatly expands both study power and the range of potential statistical methods readily available for analysis. However, national and international legislations could limit direct data sharing in the future. Therefore, the BMSD registries are also scoping a federated data analysis approach which offers the benefit of joint analysis of data across several data sources without data leaving the local sites and hence legal complexities can be avoided. This encompasses descriptive statistics as well as more advanced statistical modelling like regression analysis, then referred to as federated learning often requiring multiple iterations of analysis that need to be well coordinated and simultaneous, The challenges primarily stem from the absence of established frameworks for numerous statistical models and practical limitations, including the presence of firewalls. Consequently, further development is required to overcome these obstacles and refine this approach.

Whether using a pooled dataset or a federated approach, data need to be harmonized between the data sources and organized in a CDM. A major effort is therefore to create a more complete CDM, a work which is now being finalized and which will be published in the coming year. The basis of the CDM is the BMSD data dictionary which contains close to a hundred items with agreed upon common definitions and descriptions. In addition, we have developed a BMSD CDM software which will translate a local database into the BMSD data format and generate a report on the success of the transformation of data and a second report on the data quality in terms of data density and completeness (to be published). These tools will systematically be applied to assess quality issues within and between the BMSD registries as well as for registries seeking to join BMSD in the future.

BMSD aspires to pioneer further development of federated approaches for joint analyses of data, including federated learning, to allow more complex analyses without merging data. Furthermore, another aim of BMSD is to actively promote the standardization of definitions and procedures in MS RWE research, including PASS. This will be a gradual process. Each registry will be required to harmonize its own data to the core CDM, which can be customized according to the specific requirements of each specific project and aligned within the participating registries. Once a consensus has been reached on the harmonization process, it will serve as a foundation for developing additional analytical principles.

## Future perspectives

BMSD constitutes a network of MS registries working together since 2014 to provide an unparalleled real-world dataset for researchers, MAHs and regulatory bodies. BMSD will soon renew an application to EMA for a qualification opinion regarding PASS. If approved, this would provide standardised expectations for MS registries when participating in regulator-demanded studies as well as guidelines for registries interested in joining the BMSD network. Moving forward, BMSD aims to pursue a qualification opinion for PAES.

The notable advantage of BMSD lies in its possession of extensive, high-quality patient data, which allows the study of rare safety events, comparisons between countries and over time, and direct comparisons between different treatment exposures as the safety data is collected from all patients irrespective of DMT exposure.

Real-world data from patient registries differ inevitably from clinical trial data. Registry visits and tests are irregular, whereas trials conform to a very specific visit schedule. Source data verification is also usually not feasible or highly restricted. Further, a collaboration between registries from different populations using different IT platforms introduces dynamic heterogeneity in datasets which need to be handled by a well-developed data management routine and strong coordination of new or updated data fields.

Observational studies using MS registries provide opportunities for external validation of clinical trial data, head-to-head treatment comparisons and multi-year longitudinal assessments [12]. It can assess treatment effectiveness and safety in treated populations that are usually excluded from clinical trials, such as people under the age of 18 or over 55, or people with prior comorbidities such as diabetes, cancer or serious mental health issues. BMSD amplifies these opportunities further by its sample size, collegiate leadership, and support of a well-integrated network of statisticians and data managers. BMSD organized its first conference on statistical approaches in MS epidemiology in 2019 and a second in 2023 and has the expressed ambition to contribute to the development of this field of investigation.

It is a clear ambition of BMSD to include more MS registries in the future. Having spent time and effort to define common scope and properties, harmonize variables and definitions in a common data model which also provides means of assessing data quality and density, BMSD will expect MS registries wanting to join BMSD to prove their fit-for-purpose at a similar level as the current six registries. It is our impression that some non-BMSD MS registries are already now qualified to join, but a review has not yet been initiated.

In conclusion, a well-established network of MS registries offers substantial advantages for real-world data analysis, including comparative effectiveness and comparative safety and pregnancy outcomes studies. The very large sample size allows the exploration of causality and association for rare events. BMSD is already making valuable contributions to clinicians, researchers, MAHs, and regulators, with the ultimate aim of better outcomes for people with MS.
